# Innovative Spectrum Handoff Process Using a Machine Learning-Based Metaheuristic Algorithm

**DOI:** 10.3390/s23042011

**Published:** 2023-02-10

**Authors:** Vikas Srivastava, Parulpreet Singh, Praveen Kumar Malik, Rajesh Singh, Sudeep Tanwar, Fayez Alqahtani, Amr Tolba, Verdes Marina, Maria Simona Raboaca

**Affiliations:** 1 School of Electronics and Electrical Engineering, Lovely Professional University, Phagwara 144411, India; 2 Department of Electronics and Communication Engineering, Pranveer Singh Institute of Technology, Kanpur 208001, India; 3 Division of Research and Innovation, Uttaranchal University, Dehradun 248007, India; 4 Department of Computer Science and Engineering, Institute of Technology, Nirma University, Ahmedabad 382481, India; 5 Software Engineering Department, College of Computer and Information Sciences, King Saud University, Riyadh 12372, Saudi Arabia; 6 Computer Science Department, Community College, King Saud University, Riyadh 11437, Saudi Arabia; 7 Department of Building Services, Faculty of Civil Engineering and Building Services, Technical University of Gheorghe Asachi, 700050 Iași, Romania; 8 Doctoral School, University Politehnica of Bucharest, Splaiul Independentei Street No. 313, 060042 Bucharest, Romania; 9 National Research and Development Institute for Cryogenic and Isotopic Technologies—ICSI Rm. Vâlcea, Uzinei Street, No. 4, 240050 Râmnicu Vâlcea, Romania

**Keywords:** cognitive radio network, support vector machine, red deer algorithm, spectrum handoff, spectrum sensing

## Abstract

A cognitive radio network (CRN) is an intelligent network that can detect unoccupied spectrum space without interfering with the primary user (PU). Spectrum scarcity arises due to the stable channel allocation, which the CRN handles. Spectrum handoff management is a critical problem that must be addressed in the CRN to ensure indefinite connection and profitable use of unallocated spectrum space for secondary users (SUs). Spectrum handoff (SHO) has some disadvantages, i.e., communication delay and power consumption. To overcome these drawbacks, a reduction in handoff should be a priority. This study proposes the use of dynamic spectrum access (DSA) to check for available channels for SU during handoff using a metaheuristic algorithm depending on machine learning. The simulation results show that the proposed “support vector machine-based red deer algorithm” (SVM-RDA) is resilient and has low complexity. The suggested algorithm’s experimental setup offers several handoffs, unsuccessful handoffs, handoff delay, throughput, signal-to-noise ratio (SNR), SU bandwidth, and total spectrum bandwidth. This study provides an improved system performance during SHO. The inferred technique anticipates handoff delay and minimizes the handoff numbers. The results show that the recommended method is better at making predictions with fewer handoffs compared to the other three.

## 1. Introduction

Because of the ever-expanding number of wireless applications, the demand for wireless data has recently risen, and it is expected to continue. Compared to 2015, the monthly consumption of mobile data is predicted to increase eight times by 2022. Several studies have demonstrated that the PU underutilizes the spectrum. Spectrum gaps are unoccupied areas of range that can be used for wireless communication. The CRN is the name given to the network adopting this technology. A CRN permits the SU to access the radio spectrum of the PU on an as-needed basis, depending on the availability of the radio spectrum and with the least possibility of interference [1].

The PU has a higher priority, whereas the SU has no importance and is responsible for identifying the spectrum gaps so that it can occupy the channel without interfering with the PU’s operation. Cognitive radio comprises four primary features, referred to as the “cognitive radio cycle”: spectrum sensing, spectrum sharing, spectrum decision-making, and spectrum mobility [2]. This research focuses on the mobility phase, that is the handoff procedure. SUs must vacate their current spectrum band as soon as possible if they detect an oncoming PU. Therefore, the SU will release a channel to locate the next available channel to ensure seamless communication when a PU comes. The cohabitation of SUs with PUs is one of spectrum mobility’s most challenging and essential issues. The handoff process is critical in CRN because changing the SUs’ operational frequency range manages spectrum mobility and ensures uninterrupted SU transmission. In the absence of free channels, rerouting happens. To start with, the presence of the PU on the licensed channel effectively forces the SU to create handoffs [3].

Since the handover process relies on both the SU and the PU, the evaluation of CRN is performed by monitoring both. The most common causes of SHO are the SU’s mobility, a lack of accessible spectrum, and the identification of a PU on the channel utilized by the SU. The handoff process is cyclic; the phases are evaluation and link maintenance [4]. There are four types handoff strategies: (i) handoff triggering-timing-based [5], (ii) mobility-based spectrum aware [5], (iii) probability-based [6], and (iv) spectrum sensing-based [7]. To choose the best handoff algorithm for a certain context, it is necessary to assess its performance using a specific performance indicator. The SHO performance indicators determine the overall efficiency of CR networks. SHO performance indicators are spectrum handoffs numbers, cumulative handoff delay, link maintenance probability, effective data rate, probability of misdetection and false alarm, SHO ratio, bandwidth utilization, energy consumption, collision probability, blocking probability, and forced termination probability. Handoff algorithms are typically implemented based on the criteria for handoff and the method by which the criteria are processed. There are two main types of handoffs in the CRN: the traditional type, which is only based on handoff criteria; and the emerging type, which is based on the method of handoff criteria [8]. In traditional handoff algorithms, handoff criteria include the SNR, received signal strength, and bit error rate. The emerging category of handoff includes algorithms that are based on dynamic programming, prediction, fuzzy logic [9], neural networks [10], and deep learning [10].

A broad and dynamic spectrum is necessary to perform an opportunistic SHO. Because PUs are the licensed users of the spectrum, SUs have a lower priority in the CRN than PUs. The SU must evacuate the spectrum instantly upon the arrival of the PU without interruption in its transmission. This is extremely difficult and results in interference; thus, the SHO method should include an effective interference avoidance mechanism.

This research investigates whether decreased handoffs improve network efficiency regarding delay and power. Thus, the handoff function is meant to be reduced in optimization terms using Equation (1).

(1) $$f_{0}:\;\min(\sum h_{0}\left(t\right))$$

In this case, *h*_0_(*t*) denotes the number of handoffs in a route searched by different algorithms. A CR network is an intelligent system based on various processes such as examining, observing, planning, learning, and adapting. Hence, the learning process is a critical component that may be treated from different perspectives, including robust control approaches, evolutionary algorithms, machine learning, and artificial intelligence [11]. Machine learning and evolutionary processes have gained more recognition for solving specific issues efficiently. The learn heuristic method, a machine learning-based metaheuristic algorithm, examines spectrum sensing and handoff problems in wireless networks. This machine learning-based metaheuristic algorithm is compared to an improved particle swarm optimization (iPSO), a genetic algorithm (GA), and spectrum PSO (specPSO). Comparison is based on parameters such as the SU bandwidth, the total bandwidth of spectrum, the SNR, and throughput during handoff. The machine learning-based metaheuristic method (SVM-RDA) has been proposed [12]. SVM-RDA addresses the problem related to local minima and premature convergence in various PSO (including iPSO and SpecPSO) and GA configurations. This experimental parameter yields a more optimal result than the previous one.

This paper’s novelty is that the authors used a machine learning-based metaheuristic algorithm. Machine learning forecasters can be trained to forecast the status of the occupied and unoccupied channels with fewer handoffs. Thus, more than machine learning models are required to fulfill the precision needed for channel prediction in the cognitive radio network. Metaheuristic algorithms are applied as a search guide to obtain the near-optimal approximate solution that can increase specific systems’ performance with reasonable computing costs. The metaheuristics are used with machine learning networks for two purposes: (1) estimating and tuning the parameter of the model during a training process, and (2) tuning the hyperplane related to network structure.

The objective of this research paper is as follows:

- The handoff reduction technique of the CRN is proposed, which increases the performance of the CRN (decreased number of handoff, decreased number of failed handoffs, decreased delay, increased E_b_/N_o_, and increased throughput).
- Although metaheuristic algorithms and machine learning techniques were initially developed for different purposes, here they are integrated. To the best of our knowledge, there is no research paper on integrating machine learning techniques into metaheuristics that investigates the technical aspect of the CRN.

The structure of this article is as follows: Section 2 discusses previous work inspired by different algorithms to solve CRN problems through a literature review and problem statement. Section 3 discusses algorithms related to the research. Section 4 introduces the proposed machine learning-based metaheuristic algorithm and methodology. Section 5 presents the results of the methods described under various simulation scenarios. Section 6 delves into the topic of result analysis. Finally, in Section 7, the findings of this research are concluded.

## 2. Literature Survey and Problem Statement

Lala et al. [13] described sensing the spectrum as detecting the presence of unoccupied frequency regions in the radio spectrum. Upon the arrival of a PU, the SHO is arranged. It is suitable for cognitive users to recognize appropriate channels to resume their unfinished transmission. CR, which learns from its surroundings and is expressed using machine learning and artificial intelligence ideas for learning and reasoning. Machine learning, also known as cognitive learning, is a type of learning in which the machine records all channels and detects them by monitoring the idle state to allot to the SU. When the PU demands a channel, another unoccupied channel is discovered and switched over. Detection using a cognitive learning system is proposed when SHO is necessary. Zhang et al. [14] introduced reinforcement learning with fuzzy interference for DSA in CRN. Cognitive users’ spectrum usage is dynamically assigned, and there is no interference with the SU or the PU. Simultaneously, as an integral aspect of intelligent communication technology, sharing spectrum resources between PUs and SUs is critical. Awoyemi et al. [15] examined how DSA uses channels for the SU. DSA detects the open spectrum when the PU is absent and shares the channels with the SU. When PUs return, SUs suspends their process until it is relocated to another vacant channel. The SU resumes its function after reconfiguration to the assigned channel. Wang et al. [16] suggested a strategy for allocating the spectrum in CRNs based on reinforcement learning. DSA allows PUs to utilize the SU’s frequency and bandwidth without interfering. DSA has the potential to enhance spectrum allocation. DSA detects vacant/white space, which the SU then uses to provide a smooth transmission.

Yucek et al. [17] concentrated on the principles of cooperative spectrum sensing in the CRN. Spectrum sensing is critical in CRNs because it detects the spectrum hole that the SU uses to transmit data. A few spectrum sensing features are employed to determine interference, noise, parameter utilization, and power. Combination of two bio-inspired algorithms develop a spectrum allocation strategy for many SUs. The simulation findings demonstrate that the proposed max feeding optimization approach is resilient and straightforward, making it a more appropriate solution for the problem related to the spectrum allocation in CRN. After comparing the two algorithms, it was determined that the max feeding approach provides the best routes with the fewest handoffs. Gogoi et al. [18] projected optimizing energy usage by swarm-based intelligence algorithms such as the whale optimization algorithm, human behavior-based particle swarm optimization, particle swarm optimization with an aging leader and challengers, and particle swarm optimization (PSO). Swarm intelligence deals with combined information about artificial and natural systems. Here, PSO, specPSO, and GA swarm-based optimization algorithms are used. Dhivya et al. [19] discuss about Ingenious Method for Conducive Handoff Appliance in Cognitive Radio Networks. DSA can be used to check for available channels for the SU when handoff occurs and that an improved swarm-based cognitive radio approach be used to overcome this issue. The suggested approach’s simulation results are compared to those obtained using a GA and the SpecPSO. It is possible to achieve high data rates and high throughput using the suggested algorithm’s experimental parameter while maintaining the whole spectrum’s total bandwidth and SU bandwidth. This article demonstrates improved system performance throughout the SHO process. Dhivya et al. [20] suggested a hybrid new technique related to fuzzy rough set theory and SVM for managing handoff mechanisms. It is indicated that this approach anticipates the node in the lead where handoff should be launched to decrease handoff numbers and delay. Considering minimum handoffs, the proposed technique has improved prediction mechanisms. Empirical research explains the quantitative aspects of spectrum mobility prediction in-depth. These factors were intensively investigated since they are critical to the SHO. 

Singh L et al. [21] demonstrated a GA that raised the likelihood of achieving the best weight by optimizing network spectrum prediction with reasonable accuracy. It improves handover by using specPSO. Reinforcement learning demonstrate the effectiveness of cognitive IoT and showed that it outperforms a single-channel access model. Table 1 shows the comparison of the proposed model with the state-of-the-art models considering parameters such as Algorithm used, technique used and finally the outcome of the state-of-the-art models. 

Problem Statement: Congestion in wireless communication is caused by a lack of available radio waves, an issue addressed through the development of the CRN. The cognitive cycle’s spectrum mobility phase is critical in ensuring a seamless handover. Since the present methods do not provide smooth connectivity and do not cater to the various network needs, the proposed algorithm focuses on inventing an intelligent approach that fine-tunes and administers the handoff process before the network ambiance changes.

## 3. Algorithm

### 3.1. Genetics Algorithm (GA)

In the 1960s and 1970s, John H. Holland and his colleagues invented the GA, becoming one of the most extensively used meta-heuristic algorithms. There are three genetic operators in the Darwinian evolution principle of biological systems: reproduction, crossover, and mutation [28]. It is based on an abstract version of Darwin’s evolution principle of biological systems. Chromosomes are long strings (typically decimal or binary) that encode every solution. The value of the fitness function is calculated in each iteration. After that, the values are ordered from highest to lowest. The best answers are at the top of the list, and these are the ones that will be replicated. Low-fitness solutions are discarded.

### 3.2. Particle Swarm Optimization (PSO)

This is a population-based algorithm optimization approach. PSO’s method was initially designed to simulate birds flocking to food sources, but they efficiently solved optimization problems. PSO is an evolutionary approach like stochastic diffusion search, GA, and fuzzy logic. Particles are PUs and SUs [29].

### 3.3. SpecPSO

Visiting the location register is the best option for the local area. In contrast, the home location register is the finest option for the global zone, which is effective for SHO. SpecPSO is utilized in conjunction with “machine-learning principles” to perceive the channel. SpecPSO utilizes the notion of a learning engine that determines the condition of the channel and makes an optimal choice of using the frequency bandwidth available in the channel [29].

### 3.4. iPSO

PSO is an essential and straightforward way to handle the best local and global search. iPSO’s performance is determined by inertia weight and learning factor. Inertia weight is utilized to enhance the channel’s sensing capability. Learning aspects, including cognitive characteristics, are improved when iteration increases to advance the global search. The parameter is initialized via chaotic optimization. To cope with the study’s performance, it is necessary to apply chaotic optimization. This algorithm generates a broad variable range of integers instead of random numbers selected from the population. The PU and the SU are the components of the CRN in this technique. It is used in conjunction with spectrum sensing to keep track of free channel availability in the spectrum. The iPSO approach, based on swarm-based network optimization, is used to determine the ideal search space for the SU [30].

### 3.5. Support Vector Machine

Machine learning, a subclass of artificial intelligence, is used in the CRN. There are three types of learning: supervised, unsupervised, and reinforcement learning. Supervised learning learns from the training dataset and needs prior information about the atmosphere. Unsupervised learning does not need any training dataset, and it accomplishes self-adapting actions without prior knowledge about the atmosphere. In reinforcement learning, learning agents learn by observing the actions of other agents, and the learning regime and working atmosphere influence their performance. Since the CRN can learn radio environments through a cognitive engine, the use of machine learning algorithms can increase the reasoning and learning of the SHO process. Here, a learning model represents the process of acquiring knowledge by interacting with the environment to improve the future decisions. SVM is supervised learning. Classification and regression issues can be solved using supervised machine learning. However, it is mainly used for sorting. The SVM algorithm constructs the best decision boundary or line called a hyperplane to place consecutive data points in the correct category efficiently. Then, the hyper-plane that separates the two groups is selected. Support vectors near the hyperplane alter their location and orientation. Using these vectors, the classifier’s margin is maximized. If the support vectors are removed, the hyperplane moves. These characteristics help us design our SVM [31].

### 3.6. Red Deer Algorithm (RDA)

The RDA initializes with a random population of red deer (RD). The greatest RD in the population are labeled “male RD”, while the remaining are called “hinds”. First, the male red deer must yell. Based on the roaring strength, male RD are classified into two groups: (i) stags and (ii) commanders. Then, each commander of the harem and stags fight to possess their harem. Commanders also build harems. There are direct links between harem size and leaders’ screaming and combat ability. So, commanders’ mate with harems of hinds. Stags mate with the closest hind of the harem [32].

The roaring of male RD is the solution space equivalent to local search to increase exploitation characteristics. Similarly, combat between commanders and stags is seen as a local search; nevertheless, we only accept the better-observed answers in this process. This stage also evaluates the features of exploitation. Following that, harems are constructed and assigned to commanders based on their power. This step facilitates the exploration phase of the algorithm. Under the rules of the game, the harem’s commander mates with the proportion of hinds in his harem and the percentage of hinds in other harems. During the breeding season, all stags should mate with the hind near them, that is, a stag should mate with the hind closest to him without considering the size of the harem. This step is likewise concerned with the exploration and the exploitation phases simultaneously [33].

#### 3.6.1. Create a Beginning Group of Red Deer

In terms of the problem’s variables, optimization aims to find global or near-optimal solutions that optimize an array of variables. An example is GA; this array is referred to as a “chromosome”, whereas in the RDA, this array is referred to as an “RD”. Note that an “RD” represents a possible solution X within solution space. As a result, RD serves as a contrast to a solution. This solution X has N_var_ dimensions, which means it has N variables.

Red Deer = [X_1_, X_2_, X_3_, ...; X_Nvar_] (2)

Hence, first, a population of size N_pop_ is built to use as a starting point for the method. The finest RD is given to N_male_ and the remainder to N_hind_ (N_hind_ = N_pop_ − N_male_).

#### 3.6.2. Roar Male RD

By shouting, male RD attempt to improve their elegance. So, the roaring procedure can either fail or succeed. The male RD’s neighbors are found and replaced with preceding ones if their roaring strength (objective functions) are better than male RD’s. However, each of the male RD shift positions. Equation (3) is presented to update the status of males:

(3) $${\mathrm{male}}_{\mathrm{new}}=\left\{\begin{array}{c} {\mathrm{male}}_{\mathrm{old}}+a_{1}X\left(\left(\mathrm{UB}-\mathrm{LB}\right){\;*\;a}_{2}\right)+\mathrm{LB}\;\mathrm{if}\;a_{3}>0.5\\ {\mathrm{male}}_{\mathrm{old}}-a_{1}X\left(\left(\mathrm{UB}-\mathrm{LB}\right){\;*\;a}_{2}\right)+\mathrm{LB}\;\mathrm{if}\;a_{3}<0.5 \end{array}\right.$$

LB and UB restrict the search to find viable neighborhood alternatives for males. The upper (UB) and lower bounds (LB) can be found in the search area. The male_new_ is the most recent update to the existing position of male RD. a_1_; a_2_; and a_3_ are created by a random distribution between zero and one when it comes to the roaring process in nature.

#### 3.6.3. Select γ Percent of the Best Male RD as Male Commanders

There is a great deal of variation amongst male RD in nature. Some are stronger and more effective at expanding their area than others. As a result, RD are classified into two types: stags and commanders. Equation (4) is used to determine the number of male commanders:

(4) $$N_{\mathrm{com}}=\mathrm{round}\;\{\gamma \;*\;N_{\mathrm{male}}\}$$

The number of males, harem leaders is N_com_. The algorithm model’s starting value is γ. The range of γ is zero to one. Stags are counted in Equation (5):

(5) $$N_{\mathrm{stag}}=N_{\mathrm{male}}-N_{\mathrm{com}}$$

According to the population of males, the number of stags is N_stag_.

#### 3.6.4. A Fight between Stags and Male Commanders

They are constant fights between male commanders and stags. Stags were assigned to each commander randomly, which allowed the commander and stag to approach each other in the solution space. As a result, there are two new options and may remove the commander and install the most effective option. Two mathematical formulae are proposed for the fighting process:

(6) $$\mathrm{New}\;1=\frac{\mathrm{Com}+\mathrm{Stag}}{2}+{\;b}_{1}\;*\;\left(\left(\mathrm{UB}-\mathrm{LB}\right)\;*\;b_{2}\right)+\;\mathrm{LB}\;$$

(7) $$\mathrm{New}\;2=\frac{\mathrm{Com}+\mathrm{Stag}}{2}-b_{1}\;*\;\left(\left(\mathrm{UB}-\mathrm{LB}\right)\;*\;b_{2}\right)+\;\mathrm{LB}$$

Two new solutions are developed due to the fighting process: New 1 and New 2. Stags and commanders are both represented by the Stag and Com symbols. LB and UB limit the search space’s lower and upper bounds regarding the viability of new solutions. Here, b_1_ and b_2_ are random variables with uniform distribution. The range of b_1_ and b_2_ is between 0 and 1.

#### 3.6.5. Form Harems

The harems are formed. A harem is a group of hinds gathered by a male leader. The male commanders’ strength determines the number of hinds in harems. Hence, hinds are divided among commanders proportionally to establish the harems:

(8) $$V_{n}=v_{n}-\max_{i}\left\{v_{i}\right\}$$

where v_n_ is the nth commander’s power and its normalized value is V_n_. Thus, the commanders’ normalized power is

(9) $$P_{n}=\left|\frac{V_{n}}{\sum_{i=1}^{N_{\mathrm{com}}}V_{i}}\right|.$$

The number of hinds of a harem is

(10) $$N.{\mathrm{harem}}_{n}=\mathrm{round}\left\{P_{n}.N_{\mathrm{hind}}\right\}$$

where N.${\mathrm{harem}}_{n}$ is hinds’ number in the nth harem, and N_hind_ is the total number of hinds in the whole population. So, the hinds are distributed evenly among the male commanders by selecting N.${\mathrm{harem}}_{n}$ of the hinds at random.

#### 3.6.6. Mate Commander of a Harem with α% of Hinds in His Harem

Deer breeds like other animals. It is the commander who breeds and the deer make up α% of hinds he keeps in his harem.

(11) $$N.{\mathrm{harem}}_{n}^{\mathrm{male}}=\mathrm{round}\left\{\alpha .N.{\mathrm{harem}}_{n}\right\}$$

In this case, $N.{\mathrm{harem}}_{n}^{\mathrm{male}}$ represents the total number of harem hinds who have their leader mate with them. Here, $N.{\mathrm{harem}}_{n}^{\mathrm{male}}$ of the N.${\mathrm{harem}}_{n}$ in the solution space is randomly selected. In the model of the RDA, α is an initial parameter that serves as a starting point. It has a range from zero to one. The offspring solution is often defined as Equation (12):

(12) $$\mathrm{offs}=\frac{\mathrm{Com}+\mathrm{Hind}}{2}+\left(\mathrm{UB}-\mathrm{LB}\right)\;*\;c$$

where commanders and hinds are denoted as Com and Hind, respectively. Offs is the new solution.

#### 3.6.7. Mate Commander of Harem with β% of Hinds in Another Harem

Male commanders can mate with a percentage of hinds in a randomly selected harem (named k). They go on the offensive against another harem to expand their area. The algorithm model’s starting parameter value is β. This parameter has a value range from 0 to 1. This formula is used to estimate the harem’s total number of hinds that will mate with the commander:

(13) $$N.{\mathrm{harem}}_{k}^{\mathrm{male}}=\mathrm{round}\left\{\beta .N.{\mathrm{harem}}_{k}\right\}$$

where $N.{\mathrm{harem}}_{k}^{\mathrm{male}}$ is the number of hinds in the kth harem which mate with the commander.

#### 3.6.8. Mate Stag with the Nearest Hind

Each stag mates with its nearest hind in this phase. A male red deer is more likely to follow a hind during the mating season. This hind may be his chosen among all hinds, regardless of the harem areas. There is no restriction on which hind might be his partner. Several formulas may determine how far a stag is from every hind in J-dimension space.

(14) $$d_{i}=(\underset{jͼJ}{\sum }{\left({\mathrm{stag}}_{j}-{\mathrm{hind}}_{j}^{i}\right)}^{2}$$

where $d_{i}$ is the distance between the stag and the i^th^ hind. Accordingly, the minimum value in this matrix represents the selected hind.

#### 3.6.9. Select the Next Generation

Two distinct procedures have been used to pick the next generation. At first, we keep all of the male red deer, commanders, and stags. The second approach is concerned with the following generation’s population.

#### 3.6.10. Stopping Condition

For example, the number of iterations performed might determine the stopping condition and the quality of the most outstanding solution ever discovered.

### 3.7. SNR and Average Throughput

The SNR threshold is determined to be 15 dB using Equation (15). The throughput is computed by multiplying the number of payloads by the total time it takes to complete the transmission using Equation (16).

(15) $${\mathrm{SNR}}_{\mathrm{db}}=10{\;\log}_{10}\frac{\mathrm{Signal}\;\mathrm{power}}{\mathrm{Noise}\;\mathrm{power}}$$

(16) $$\mathrm{Average}\;\mathrm{throughpt}=\frac{\mathrm{Total}\;\mathrm{payload}}{\mathrm{Time}}$$

## 4. Methodology

This section aims to familiarize the reader with the algorithms and demonstrate how they may be applied in CRN issues by metaheuristic methods, such as GA, PSO, cuckoo search, max feeding optimization, ant colony optimization, and artificial bee colony, may be found in the contemporary literature. The SVM and RDA were chosen for this inquiry because they have demonstrated the most outstanding performance in cases comparable to identifying a spectral opportunity in CRNs [34].

### SVM-RDA

**Figure 1:**
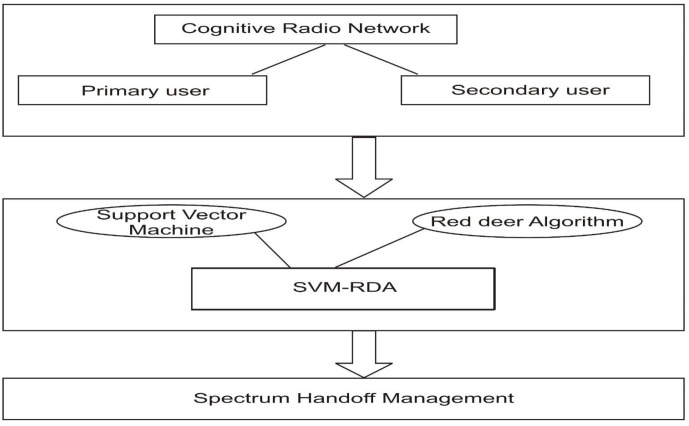
Proposed architecture diagram of SVM-RDA.

SVM-RDA-based SHO decision-making makes the CRN handoff procedure more efficient. The hybrid SVM-RDA and the CRN are used to assess network metrics such as the SNR, throughput, number of handoffs, and number of failed handoffs. This approach allows the SU to move for an unoccupied channel in advance and reduce connection failures. This hybrid handoff technique assures accurate and predictive judgments by considering all possible outcomes, even when the environment is unknown. The workflow is separated into two stages [35]. The first section includes preprocessing and SVM training data, and the second section incorporates the RDA for optimizing SVM output. In this method, CRN has PUs and SUs. From spectrum sensing, here the availability of free channels is obtained. The machine learning-based metaheuristic optimization technique SVM-RDA is used to find the optimal results for the SU, as discussed in Figure 1.

**Figure 2:**
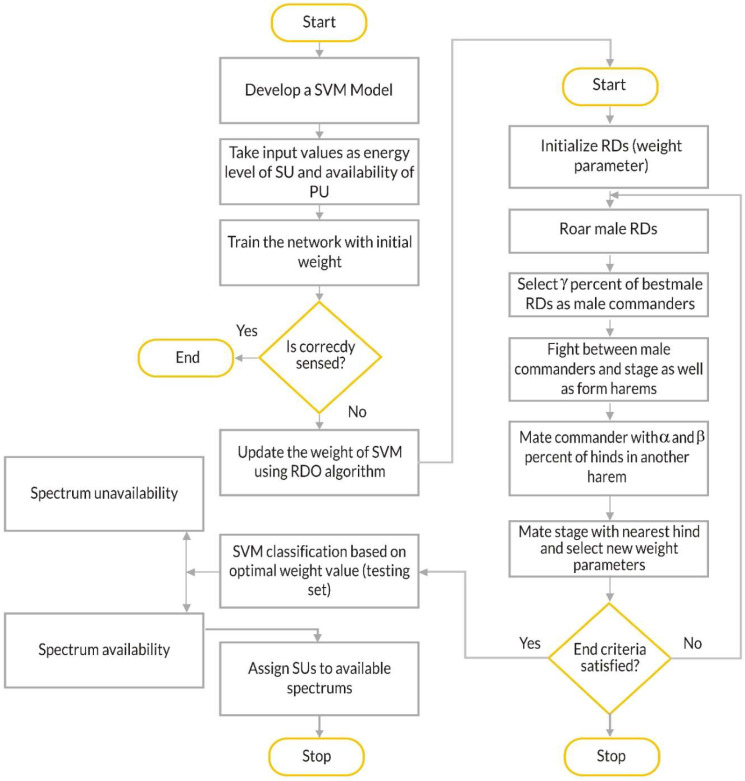
Flowchart of SVM-RDA.

A flowchart describing the workflow of the proposed SVM-RDA algorithm is shown in Figure 2.

## 5. Results

### 5.1. Parameter Configuration for the SVM-RDA Algorithm

The method used to optimize the process significantly impacts the selected parameters. Table 2 shows the suggested approach’s parameter configuration used in the simulation test. Analysis of handoff parameters is made based on the handoff methods, including the GA, SpecPSO, and iPSO.

### 5.2. Setup for Experiment

The suggested approach is evaluated in terms of performance using Matlab R2014a on a 64-bit computer equipped with core i7 CPU and 8 GB RAM. The simulation environment consists of 20 nodes placed in an area of 500 × 500 square meters for 1000 s of simulation time. The population size is set to 50, and the iteration number is changed between 0 and 100. User count is estimated to be 50, and the number of channels available is considered to be 200. Bandwidth is configured to be 30 kHz. The maximum data transfer rate is 256 kilobits per second. Table 3 summarizes the simulation results for the spectrum and bandwidth assigned to the SU during transmission. The total dataset simulated here is 820. They range between 10 and 50 for several possible combinations of the CRN. Out of these datasets, for testing purposes, we use 164 datasets, and for training purposes, we use 656 datasets.

SNR, delay, throughput, number of handoffs, and number of unsuccessful handoffs are metrics used to monitor the handoff process. The SNR reflects how stronger a signal is compared to noise. Throughput is the total quantity of data delivered from a source node to a destination node over a particular time; data transfer must occur with minimum packet loss. The term “delay” refers to the time it takes for data to travel from a source node to a destination node. The number of handoffs refers to the number of changes in the state of channels used to send and receive data in the nodes. This quantity should be kept to a minimum to ensure uninterrupted data transfer. Failed handoff numbers refer to failed transmission numbers in a specific channel state. The system’s efficiency decreases as the number of failed handoffs increases. Here, the hybrid channel selection approach is used that includes an underlay and overlay channel model.

### 5.3. Channel Allocation of the SU

**Figure 3:**
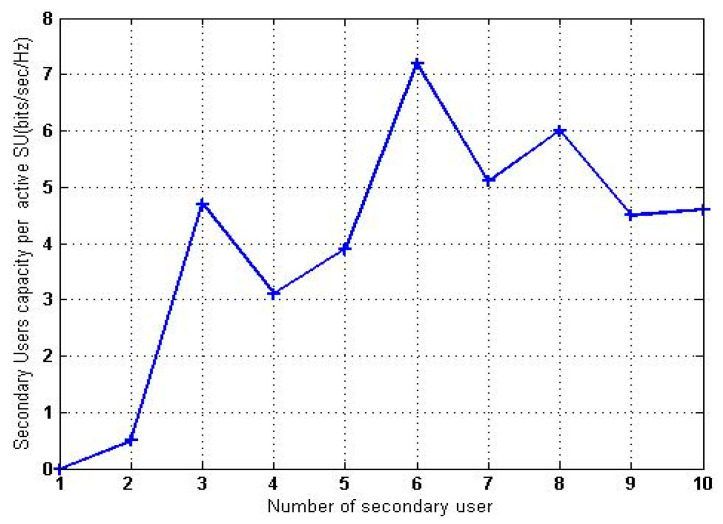
Active SU.

The CRN is used to allocate PUs and SUs. Upon the arrival of the PU, the SU must transition to the channel’s next unused frequency bandwidth. Thus, SVM-RDA determines the channel with the fewest possible repetitions, and a handoff is provided to the SU to ensure uninterrupted data transfer. As seen in Figure 3, the number of active SUs in the spectrum is noted.

**Figure 4:**
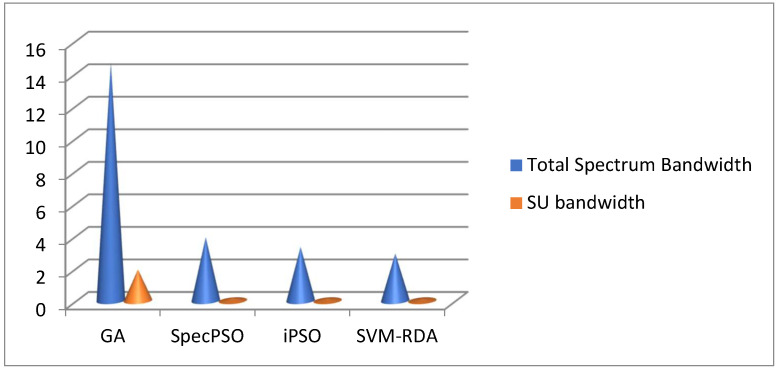
Frequency allocation for SU.

The complete PU bandwidth is measured, and the remaining SU frequency is estimated, yielding a satisfactory performance result using SVM-RDA. Figure 4 shows the simulation results for the overall spectrum and the SU bandwidth during transmission.

**Figure 5:**
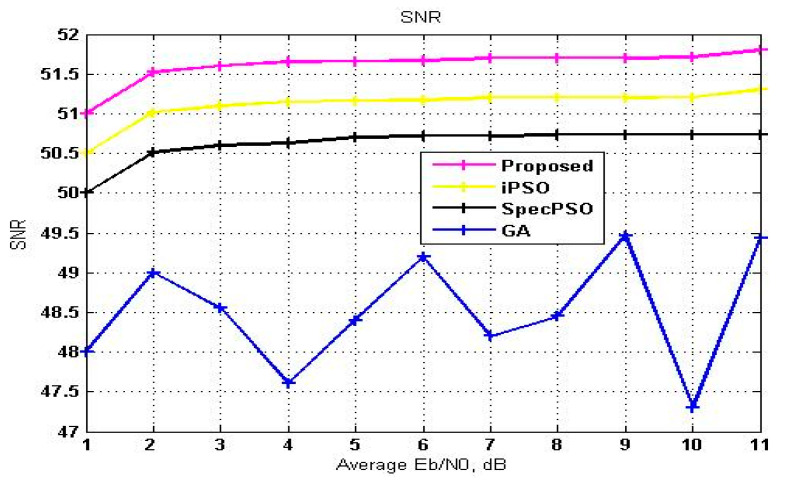
SNR vs. average E_b_/N_o_.

Figure 5 represents the SNR as a function of average E_b_/N_o_. RF frequencies are used to determine the SNR. The comparison graph suggests that the proposed method has favorable trade-off among the other three algorithms, as illustrated in Figure 5, which shows how signal is more vital than noise and interference during PU detection. E_b_/N_0_ varies from 1 to 11 dB.

**Figure 6:**
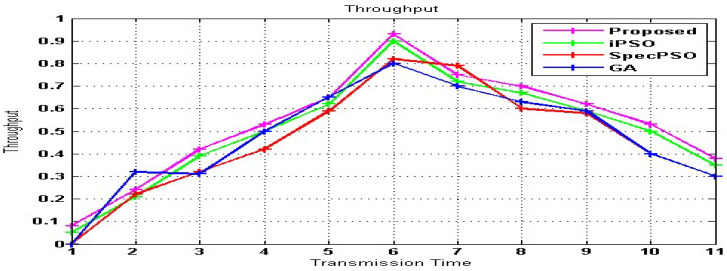
SU’s throughput after the handoff.

Figure 6 represents the throughput corresponding to our proposed model SVM-RDA, iPSO, SpecPSO, and GA as a function of transmission time. Compared to current approaches such as iPSO, GA, and specPSO, the simulation results reveal that the throughput attained is high with a faster transmission time following SU handoff, as shown in Figure 6. When the existing approaches of GA throughput = 0.8, specPSO throughput = 0.81, and iPSO throughput = 0.9 are compared to the suggested SVM-RDA throughput of 0.92, the data is improved by 22%. The transmission time ranges from 1 to 11 s. In the SVM-RDA, each SU selects a spectrum gap and transmits a single data packet within the gap. If no spectrum gap is detected, the SU would keep the data packet in its buffer for the next sensing cycle. Sending more than one packet will increase the throughput. SVM-RDA has a low complexity and a fast convergence.

**Figure 7:**
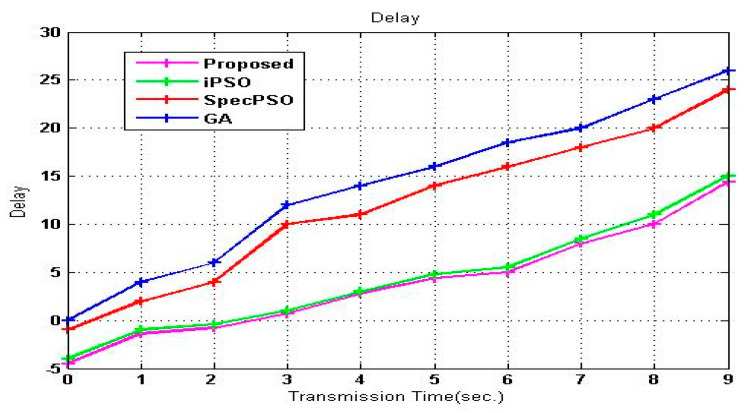
Delay vs. transmission time.

Figure 7 illustrates the delay parameter’s relationship to time. Transmission time is changed, and the delay graph demonstrates that the suggested SVM-RDA approach significantly reduces the delay compared to alternative hybrid handoff strategies. Afterward, iPSO displays the second-lowest number, followed by SpecPSO and GA. The increase in latency is primarily due to the fierce competition for channel access on these devices. The suggested approach exhibits the slightest delay since the waiting time is minimized, and SUs have immediate access to the channels.

**Figure 8:**
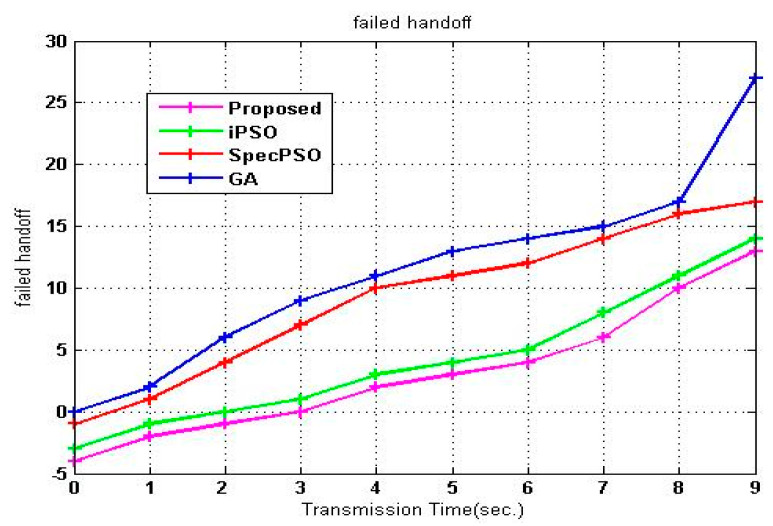
Failed handoff vs. transmission time.

The unsuccessful handoffs are depicted in Figure 8, and it is immediately apparent that the suggested SVM-RDA method has the lowest number of failed handoffs of the given algorithms tested. The iPSO algorithm displays the lowest minimum states, followed by the SpecPSO and GA algorithms. These algorithms demonstrate an improvement in unsuccessful handoffs due to the poor prediction and limited channels available for the SU to access. A significant reduction in the frequency of unsuccessful handoffs is achieved in the suggested model due to link failure prediction before range failure, which results in a more exact prediction regarding channel availability in the network and the occurrence of handoff.

**Figure 9:**
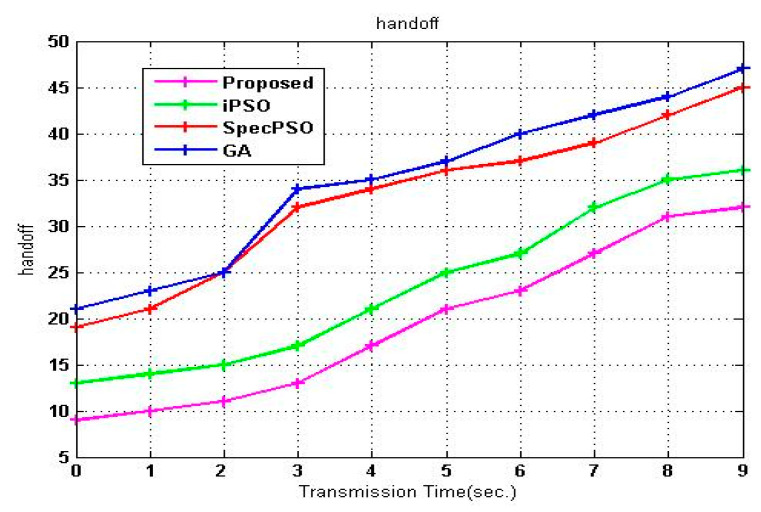
Handoff versus transmission time.

The SVM-RDA approach has a much lower number of handoffs than other algorithms. This minimum value comes from SVM- RDA optimization. These RDAs use prediction and classification before deciding which leads to this minimum value. Handoffs are minimized by intelligently predicting the handoff occurrence based on the anticipated data delivery time of the PU, as shown in Figure 9.

Thus, from the overall analysis of different hybrid handoff methods suggested in this work, the SVM-RDA handoff model is more tenable and productive in terms of the handoff parameters, thereby promoting the spectrum mobility phase in the CRN.

## 6. Result Analysis

Table 4 and Table 5 demonstrate the effectiveness of each optimization algorithm (SVM-RDA, iPSO, SpecPSO, and GA) in average delay, average throughput, failed handoff, and handoff in different transmission times values.

At a particular transmission time value (5 s), average delay is 19 s, 16 s, 8 s, and 7.8 s for GA SpecPSO, iPSO, and SVM-RDA, respectively. Hence, the proposed SVM-RDA has 58%, 51.25%, and 2.5% less average delay than GA, SpecPSO, and iPSO, respectively. At a particular transmission time value (5 s), throughput is 0.65, 0.59, 0.62, and 0.67 GA for SpecPSO, iPSO, and SVM-RDA, respectively. So, the proposed SVM-RDA has 3.07%, 10.17%, and 8.06% more throughput than GA, SpecPSO, and iPSO, respectively. At a particular transmission time value (5 s), the number of handoffs is 30, 29, 16, and 12 for GA SpecPSO, iPSO, and SVM-RDA, respectively. So, the proposed SVM-RDA has 60%, 58.62%, and 25% less handoff than GA, SpecPSO, and iPSO, respectively. The number of failed handoffs at a particular transmission time value (5 s) is 16, 15, 8, and 7 for GA, SpecPSO, iPSO, and SVM-RDA, respectively. Hence, the proposed SVM-RDA has 56.25%, 53.33%, and 12.5% less failed handoff than GA, SpecPSO, and iPSO, respectively.

Table 4 summarizes the various handoff strategies studied in network metrics. GA scheme analysis reveals that it is an ineffective handoff approach. Encapsulating the SVM-RDA demonstrates a dependable handoff scheme due to the model’s design that taking into account the disadvantages of the other system. Compared to the current iPSO, SpecPSO, and GA, the proposed algorithm is effective in SHO. Due to less handoff for spectrum mobility in the CRN, it obtained energy consumption. The RDA algorithm provides a wide usage of attributes for handoff decisions, and SVM acts as an optimization method to ensure accurate prediction.

## 7. Conclusions and Future Work

The suggested handoff strategy uses a machine learning-based metaheuristic algorithm to solve the handoff process of the spectrum mobility phase, which plays a critical role and is one of CRN’s distinctive properties. The efficiency of SVM-RDA is based on prior knowledge of the environment and reduced execution time to complete the task. The proposed strategy successfully increased the channel’s practical usage for SU. This article uses the suggested algorithm SVM-RDA to implement channel bandwidth and total bandwidth assigned for SUs, the SNR, throughput, number of handoffs, and number of failed handoffs. SVM-RDA outperforms other swarm intelligence algorithms, such as GA, SpecPSO, and iPSO. The focus of this research is mainly on the improvement of network communication via the CRN in assigning channels to the SU. Experimental findings demonstrate that the SVM-RDA algorithm ensures a seamless handoff procedure with a minimal backlog and maximizes data transfer within the specified duration. A recommendation for future work is to conduct an evaluation and validation with the most relevant autonomous learning algorithms, such as the use of binary PSO with SVM.

## Figures and Tables

**Table 1:** Comparison of proposed model with state of the art.

Research Paper	Algorithm	Technique	Outcome
Proposed	SVM-RDA	Use machine learning-based metaheuristic algorithm	Decreased number of failed handoffs, number of handoffs, and delay. Increased throughput.
Feng et al. [22]	Cognitive learning algorithm	Use machine learning and artificial intelligence-based idea	Derived average handoff time of the SU and decreased average handoff time.
David et al. [23]	Max feeding optimization algorithm	Bio-inspired algorithm	Only the problem of spectrum mobility is considered. Achieves a better handoff reduction rate.
Devi et al. [24]	Improved particle swarm optimization	Stochastic optimization technique based on swarm	Increased SNR and throughput. Decreased transmission time.
Anandakumar et al. [25]	Social cognitive handover with spectrum PSO	Use a social cognitive radio network	Effective spectrum usage and increased data transfer rate at reduced power.
Dhivya et al. [26]	Fuzzy rough set theory with SVM	Use fuzzy machine learning	Reduced handoff delay time and number of handoffs.
Babu et al. [27]	Galactic swarm optimization algorithm	Reinforcement learning	Reduced handoff delay, waiting delay, packet error rate, and service time.
Supraja et al. [28]	Genetic algorithm	Metaheuristic inspired algorithm	Reduced energy consumption and delay.

**Table 2:** Parameter configuration.

Parameter	Value
Channel type	Wireless channel
MAC type	802.11
Radio propagation model	Two ray ground
Number of channels	11
Number of PU users	10
Simulation time	1000 s
Simulation area	500 × 500 m^2^
Population size	50

**Table 3:** Bandwidth allocation for the secondary user.

Parameter	Algorithm			
	Genetic Algorithm	Spectrum Particle Swarm Optimization	Improved Particle Swarm Optimization	SVM-RDA
Total spectrum bandwidth	14.6582	3.9116	3.3202	2.1201
Secondary user bandwidth	1.9292	0.1728	0.1544	0.1123

**Table 4:** Comparison of the SNR on different average E_b_/N_0_ values for various handoff algorithms.

Average E_b_/N_0_	GA	SpecPSO	iPSO	SVM-RDA
1	48	50	50.5	51
2	49	50.51	51.02	51.52
3	48.55	50.6	51.1	51.6
4	47.66	50.63	51.15	51.65
5	48.40	50.7	51.16	51.66
6	49.2	50.72	51.17	51.67
7	48.4	50.72	51.2	51.7
8	48.45	50.73	51.2	51.7
9	49.47	50.73	51.2	51.7
10	47.3	50.73	51.21	51.71
11	49.44	50.73	51.3	51.8

**Table 5:** Comparison of handoff algorithms.

Parameters	Transmission Time (Second)	GA	SpecPSO	iPSO	SVM-RDA
Average delay in sec	1	5	4	1	0.5
	2	9	7	4	3.6
	3	11	9	4.6	4.2
	4	17	15	6	5.7
	5	19	16	8	7.8
	6	21	19	9.8	9.4
	7	23.5	21	10.5	10
	8	25	23	13.5	13
	9	28	25	16	15
Average throughput	1	0	0	0.05	0.08
	2	0.32	0.22	0.21	0.24
	3	0.31	0.32	0.39	0.42
	4	0.5	0.42	0.5	0.53
	5	0.65	0.59	0.62	0.67
	6	0.8	0.82	0.9	0.93
	7	0.7	0.79	0.72	0.75
	8	0.63	0.60	0.67	0.70
	9	0.59	0.58	0.59	0.62
Number of handoffs	1	16	14	8	4
	2	18	16	9	5
	3	20	20	10	6
	4	29	27	12	8
	5	30	29	16	12
	6	32	31	20	16
	7	35	32	22	18
	8	37	34	27	22
	9	39	37	30	26
Number of failed handoffs	1	5	4	2	1
	2	7	6	4	3
	3	11	9	5	4
	4	14	12	6	5
	5	16	15	8	7
	6	18	16	9	8
	7	19	17	10	9
	8	20	19	13	11
	9	22	21	16	15

## Data Availability

Data sharing does not apply to this article as no datasets were generated or analyzed during the current study.
